# Glutathione in HIV-Associated Neurocognitive Disorders

**DOI:** 10.3390/cimb46060330

**Published:** 2024-05-31

**Authors:** Thomas Erdos, Mika Masuda, Vishwanath Venketaraman

**Affiliations:** College of Osteopathic Medicine of the Pacific, Western University of Health Sciences, Pomona, CA 91766, USA; thomas.erdos@westernu.edu (T.E.); mika.masuda@westernu.edu (M.M.)

**Keywords:** glutathione, neuroAIDS, HIV, AIDS, HIV-associated neurocognitive disorder (HAND)

## Abstract

A large portion of patients with Human Immunodeficiency Virus (HIV) have neurologic sequelae. Those with better-controlled HIV via antiretroviral therapies generally have less severe neurologic symptoms. However, for many patients, antiretrovirals do not adequately resolve symptoms. Since much of the pathogenesis of HIV/AIDS (Autoimmune Deficiency Syndrome) involves oxidative stress either directly, through viral interaction, or indirectly, through inflammatory mechanisms, we have reviewed relevant trials of glutathione supplementation in each of the HIV-associated neurocognitive diseases and have found disease-specific results. For diseases for which trials have not been completed, predicted responses to glutathione supplementation are made based on relevant mechanisms seen in the literature. It is not sufficient to conclude that all HIV-associated neurocognitive disorders (HAND) will benefit from the antioxidant effects of glutathione supplementation. The potential effects of glutathione supplementation in patients with HAND are likely to differ based on the specific HIV-associated neurocognitive disease.

## 1. Introduction

The neurological complications seen in patients with Human Immunodeficiency Virus (HIV) and acquired immunodeficiency syndrome (AIDS) are, overall, difficult to treat and manage. The umbrella term referring to these diseases is HIV-associated neurocognitive disorder (HAND). Although HAND refers to numerous diseases, they are often spoken of as a collective. There are similar mechanisms at play between some but not all disease states of HAND. For example, in severe cases of HAND, the blood–brain barrier is often compromised. HIV-associated diseases also differ from their non-HIV counterparts. For example, HIV-associated dementia often presents in young people, unlike other typical neurologic dementias (e.g., Alzheimer’s, Lewy Body, vascular).

HAND also includes all of the opportunistic infections and malignancies that result in motor and cognitive dysfunction in HIV patients [1]. The prevalence of HAND is estimated to be between 43% and 52% among all patients living with HIV (PLWH), while less debilitating forms of HAND remain fairly common, with 33% of HIV patients having “Asymptomatic Neurocognitive Impairment” and 12% having “Mild Neurocognitive Impairment” [2,3,4]. One study by Wang et al. in 2020 estimated approximately 16 million cases worldwide of HAND in HIV-infected adults, with 72% of these cases in Sub-Saharan Africa [2]. The prevalence of various opportunistic infections, malignancies, and psychiatric disorders will be discussed later in this text.

Patients with HAND have symptoms varying from mild cognitive impairment and peripheral neuropathy to severe dementia and psychosis. Most symptoms improve as CD4+ T Cell counts rise secondary to antiretroviral therapy (ART), but some antiretroviral therapies carry side effects of neurotoxicity (e.g., efavirenz). The last two decades have been riddled with evidence of HAART therapy failing to improve or even worsening neurocognitive symptoms, despite efforts to try therapies that are better at penetrating the CNS and efforts to adjust for comorbidities [4,5,6]. Additional treatments typically included neuroprotective agents, symptom management, and supportive care.

We will discuss the potential efficacy of glutathione supplementation in each of the following HIV-related neurodegenerative diseases: HIV-associated dementia, HIV peripheral neuropathy, lymphoma, neurosyphilis, brain infection and encephalitis (due to viral, fungal, and parasitic modes), vacuolar myelopathies, and psychological conditions (including depression, anxiety, post-traumatic stress disorder, substance use disorder, sleep disturbances, and psychosis).

There have been very few trials (whether human or animal) directly testing the effects of liposomal glutathione (L-GSH) supplementation in patients with HAND [7,8]. Given a lack of data, we have extended our review to include trials involving treatments that use similar mechanisms to those seen in L-GSH supplementation. These drugs include N-acetylcysteine (NAC) (a well-known prodrug of GSH), S-adenosylmethionine (SAM) (another prodrug of glutathione), LCysSG (a new prodrug of GSH tested only in mice so far), alpha-lipoic acid (ALA), L-cysteine, methionine, and whey protein. These treatments will be discussed in the sections pertaining to the diseases in which they were trialed.

Another new prodrug of GSH is N-acetylcysteine amide (AD-4 or NACA) which comes from the amidification of a carboxyl group on NAC [9]. AD-4 has better CNS penetration than NAC [10]. It has not been tested in any HIV neurocognitive disorders. The best-understood mechanism of alpha-lipoic acid is increasing GSH [11]. L-cysteine, methionine, and cysteine-rich whey protein are simply precursors involved in the synthesis of glutathione. Deficiencies in any of these precursors can result in decreased levels of glutathione.

Many of the HIV-associated neurocognitive disorders have no trials related to glutathione supplementation. In these diseases, we make a very brief statement regarding our opinion on the potential benefit of glutathione or glutathione-related supplementation based on the current literature and understanding of the pathogenesis and mechanisms involved in the perpetuation of the disease.

We have not found any studies showing adverse effects from L-GSH supplementation. In many situations for which L-GSH is used, native GSH levels are low and exogenous treatments restore native levels. Very little foreign material is added in the process, so the expectations of adverse effects are low. In other diseases, such as diabetes, L-GSH supplementation is aimed at taking advantage of the properties of GSH rather than merely repleting its levels. These patients tend to end up with higher GSH levels than healthy subjects. The main concern here is that the increased resistance to oxidative stress may allow cancer to grow.

However, long-term L-GSH supplementation has not shown any links to cancer growth. The patients most studied in long-term trials are type II diabetics who end up with elevated GSH, decreased A1cs, increased fasting insulin levels, and few reports of adverse effects. For example, a 6 month study conducted on patients aged 30–78 years with type II diabetes mellitus found long-term supplementation to be safe with no dropouts due to adverse effects while still being beneficial to A1c and fasting insulin levels, and even more so in those over age 55 [12]. The concerns that we have for safety with glutathione supplementation in HAND are related to glutathione’s interaction with certain diseases rather than as an inherent characteristic of the drug.

As a brief reminder, HIV remains a global epidemic and public health crisis. Worldwide, approximately 39 million people were diagnosed with HIV at the end of 2022, with an estimated two-thirds, or 25.6 million, of these cases being present in the WHO (World Health Organization) African region [13]. When the supply chain is disrupted or inadequate, these individuals tend to be the first to lose care. We are interested in investigating alternative, affordable treatments for these individuals with HIV and HIV-related diseases, including HAND, as patients seem to have poor adherence to HAART secondary to availability and cost [14], with yearly costs for HAART in Africa estimated as from USD 181 to USD 730. The cheaper cost represents estimates via local manufacturing. It is possible that the local production of liposomal glutathione in sublingual or oral form may be a cheaper alternative than HAART production, so we feel it is worth researching. Perhaps L-GSH could serve as a bridge between HAART in times of drug scarcity. We are interested in discussing whether it is worthwhile to research treatment options for HAND other than HAART. In countries such as South Africa, in which the implementation of free HAART seems to be cutting mortality rates [15,16,17], it is worthwhile to see if there are any diseases for which HAART therapy does not seem to be effective and for which other treatment options should be studied further.

## 2. Impact of HIV on the Brain

Since many HIV-associated neurocognitive disorders involve infection of the brain, we will discuss in moderate detail the steps required to infect the brain and the blood–brain barrier.

### 2.1. HIV: Accessing the Cell

HIV enters host cells through a multistep process. Initially, the viral envelope protein gp120 binds to CD4 receptors on the target cell surface, followed by interaction with a co-receptor, either CCR5 or CXCR4. This binding triggers the fusion of the viral envelope with the cell membrane, releasing the viral capsid into the cell. One specific part of gp120, the V3 loop, has been found to be necessary to bind with the target cell membrane [18]. Within the cell, the viral RNA genome is reverse-transcribed into DNA, which integrates into the host cell’s chromosomal DNA. Integrated viral DNA serves as a template for the transcription and translation of viral proteins. Newly synthesized viral components are assembled into mature virus particles that bud from the host cell surface. These mature virions are capable of infecting other cells, perpetuating the viral life cycle [19].

### 2.2. HIV: Accessing the Brain

HIV attacks the brain from many directions. One of the earliest found mechanisms is through the formation of giant cells leading to HIV encephalitis [20]. When a monocyte is infected with HIV, it releases HIV-1 viral proteins such as Tat and gp120. Tat readily crosses the blood–brain barrier at human physiologic temperature (37 °C) in vitro [21]. Gp120 crosses the blood–brain barrier after the blood–brain barrier has been compromised by any of numerous mediators, including gp120 itself. Gp120 alters the expression of the tight junctions within the blood–brain barrier, resulting in increased permeability [22]. Bradykinin, a member of the kinin-kallikrein system, is well known to increase blood–brain barrier permeability and has been used in vitro to produce an increase in brain permeability greater than that seen by mannitol, which allows for the increased trafficking of monocytes into the blood–brain barrier [23]. Bradykinin is released by mast cells as part of an immune response, including as part of the typical response to HIV infection [24]. Bradykinin appears to increase permeability at least in part by decreasing the occludins and claudin-5 found in tight junctions of the barrier [25]. Once the monocytes have entered the central nervous system, they infect periventricular macrophages, microglia, and astrocytes [26]. The macrophages release HIV proteins and cytokines into the surrounding brain tissues, resulting in immune reactions. One such reaction involves the formation of nodules around perivascular macrophages by affected microglia. These nodules attach to the macrophage and, in effect, isolate it from the rest of the brain tissue. These fusions of microglia nodules and periventricular macrophages are “giant cells” (also known as “macrophage-tropic HIV virus” or “M-tropic virus”). These nodule-macrophage clumps release HIV viral proteins causing inflammation and neuronal dysfunction such as gp120, gp41, Tat, Nef, Ref, Vpr, which results in the development of HIV encephalitis [27]. This general mechanism of transport beyond the blood–brain barrier is how HIV leads to central nervous system (CNS) disease. HIV within the CNS will evolve separately from HIV outside the CNS, resulting in potentially varied symptoms and conditions that may not respond to treatments as expected.

### 2.3. Destruction of the Blood–Brain Barrier

The blood–brain barrier (BBB) is made up of tightly connected endothelial cells lining brain capillaries, along with pericytes, astrocyte end-feet, and a basal lamina. This barrier tightly controls what substances can pass from the bloodstream into the brain tissue, preventing harmful molecules from entering while allowing essential nutrients to pass through. HIV breaks down the blood–brain barrier by attacking the endothelial cell, the tight junctions between endothelial cells, and/or the astrocyte foot processes [28]. Interestingly, some ART regimens have also been associated with an increased oxidative burden within astrocytes that may affect their ability to maintain the impermeability of the blood–brain barrier [29].

#### 2.3.1. Endothelial Cells

HIV increases the permeability of the blood–brain barrier through direct destruction of its components. HIV has a Tat gene that is responsible for producing Tat protein, which increases the speed of viral transcription. The production of Tat within a perivascular macrophage activates matrix metalloproteinase 3 (MMP-3) and matrix metalloproteinase 13 (MMP-13). These matrix metalloproteinases increase PAR-1 through unclear mechanisms in HIV. In other diseases, MMPs can activate PAR-1 by cleaving proteins that release activators of PAR-1 or by cleaving the N-terminus of PAR-1. The activation of PAR-1 leads to the increased mRNA expression of monocyte chemoattractant protein 1 (MCP-1, also known as CCL2), which directly destroys endothelial cells in the blood–brain barrier [30,31]. Matrix metalloproteinases promote axonal growth, cartilage ossification, and adipose differentiation [32]. Two redox-responsive transcription factors on HIV-1 are NF-κB and AP-1 increase Tat expression. CCL2 expression can be increased by Tat and regulated by NF-κB and AP-1 [33].

#### 2.3.2. Tight Junctions

**Figure 1:**
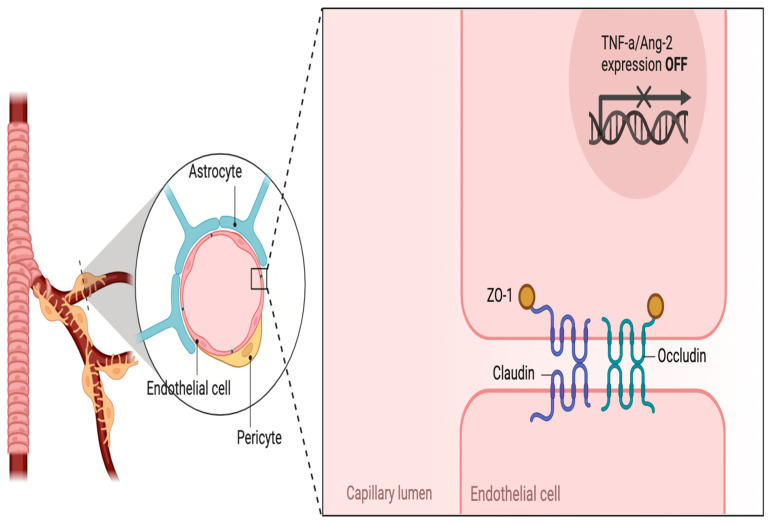
Components of the blood–brain barrier.

HIV viral proteins trigger signaling pathways that lead to the internalization of occludins and claudins (tight junction proteins), compromising the integrity of the endothelial cell tight junctions [34]; see Figure 1. A similar process is seen in the invasion of HIV beyond the brain–retinal barrier. In the eye, gp120 binds dendritic cell-specific intercellular adhesion molecule 3-grabbing non-integrin (DC-SIGN) expressed on retinal pigmented epithelium cells, resulting in the induction of metalloproteinases which disrupt tight junctions [35]. As expected, when mitochondrial GSH stores in the retina are depleted, retinal damage occurs secondary to oxidative processes [36].

#### 2.3.3. Astrocyte End-Feet

HIV can disrupt the morphology, function, and distribution of astrocyte end-feet processes, which can greatly increase the permeability of the blood–brain barrier. Part of the dysfunction within the astrocytes includes abnormal regulation of lipoxygenase/cyclooxygenase and high-conductance calcium-dependent potassium channels (BK_Ca_ channels), as well as ATP receptor activation within astrocytes [37].

### 2.4. HIV Reservoirs

As HIV is treated and managed well with ART, viral loads decrease. While HIV in the early stages of infection primarily targets lymphoid tissues such as lymph nodes, spleen, and gut-associated lymphoid tissue, it can also establish reservoirs in peripheral blood and tissues throughout the body as well as in the brain [38]. Initial access beyond the blood–brain barrier is as discussed previously and portrayed in Figure 1. The peripheral reservoirs can include cells such as monocytes, dendritic cells, and macrophages [38]. The CNS reservoirs are widely considered to be within astrocytes [39].

A study in 2020 sought to provide convincing evidence that astrocytes acted as the primary CNS reservoir for latent HIV. In the study, HIV-infected astrocytes were established by meeting the following criteria: GFAP positive, DAPI pos, presence of Alu-repeats, and presence of HIV-Nef DNA [40]. Astrocytes within brain cortical tissue, both in vitro and in vivo, were found to be infected with HIV in patients. Astrocytes are able to be used as a reservoir due to their long half-life of months to years [39]. The other infected cell types discussed, perivascular macrophages and microglia, have half-lives of 3 months versus half-lives of months to years, respectively [39]. Microglia have also been suggested as a possible reservoir of HIV-1 [26].

### 2.5. Reservoir Reactivation

In the CNS, HIV in the reservoirs can be reactivated, and new virions will be produced. Cell-to-cell contact is required for viral transfer from an astrocyte reservoir to another cell [40]. The reactivation of latent HIV in astrocytes can occur due to inflammatory signals, neuroinflammation, cellular stress, or crosstalk with neighboring cells. So far, the only drugs that have been effective in removing HIV from astrocytes are indinavir and nelfinavir [41]. In the CNS, reactivation results in many reactions, including (1) microglial repopulation; (2) increased phagocytic activity, which removes debris; and (3) astrocyte hypertrophy, which increases blood–brain barrier permeability [42].

In the periphery, various stimuli can trigger the reactivation process. These stimuli include immune activation, inflammatory signals, and disruptions in tissue homeostasis. Activation produces an infectious virus, which contributes to viral persistence and the potential resurgence of systemic infection despite antiretroviral therapy.

### 2.6. Inflammatory Processes

Many of HIV’s negative effects on the brain occur through mechanisms involving inflammation. HIV’s envelope protein, gp120, is one of the main mediators of the neuroinflammation seen in HIV patients, resulting in the potentiation of an NMDA receptor and the resultant loss of excitatory synapses [43]. The potentiation is instigated when there is basal suppression of inhibitory synapses [43]. HIV can also inhibit GABA_A_ receptors when gp120 binds to CXCR4 on microglia, causing it to release IL-1β. When IL-1β binds to IL-1 receptors on neurons, GABA_A_ receptors are inhibited [44].

It is worth mentioning, as we begin to discuss the role of certain antioxidants as treatment options to be investigated for HAND, how we might be able to determine those individuals in need of treatment. Aside from the signs and symptoms of HAND, there has been discussion of biomarkers of oxidative processes. Some of these biomarkers include MDA, acrolein, protein carbonyls, 3-nitrotyrosine, 8-hydroxy-2′-deoxyguanosine, 8-nitroguanine, malonaldehyde, and F2-Isoprostanes [45,46].

## 3. Brief Overview of Glutathione

### 3.1. Synthesis

**Figure 2:**
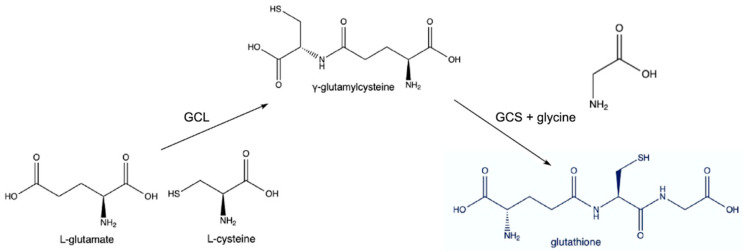
Synthesis of glutathione from glutamate, cysteine, and glycine.

Glutathione (GSH), a tripeptide composed of glutamate, cysteine, and glycine, maintains redox homeostasis and protects against oxidative stress by scavenging reactive oxygen species (ROS) and detoxifying harmful compounds. The synthesis of glutathione primarily occurs in two steps. First, glutamate and cysteine are combined by the enzyme γ-glutamylcysteine ligase (GCL) to form γ-glutamylcysteine. Then, γ-glutamylcysteine is combined with glycine by glutathione synthetase to produce glutathione; see Figure 2. This process is regulated by the availability of substrates, particularly cysteine, which is often the rate-limiting factor due to its relative scarcity [47].

### 3.2. Antioxidant Mechanisms and Neuroprotection

Since GSH acts directly on reactive oxygen series (ROS) and reactive nitrogen series (RNS), it requires proximity to provide protection. If the storage of GSH in the brain is insufficient, then there is a risk for oxidative stress, particularly in the areas in which GSH is deficient. For example, astrocytic Nrf2 is activated by oxidative stress and upregulates astrocytic GSH, which protects neurons against oxidative stress [48,49,50]. Astrocytes release GSH at a rate of about 10% of their total intracellular store per hour [51]. In addition, microglial GSH protects against oxidative stress in pathologic causes within the brain [51].

There are cellular processes that also create ROS and RNS. Part of synthesizing ATP inside mitochondria involves the production of ROS [52], so mitochondria need to be able to manage the ROS effectively. GSH is synthesized in the cytoplasm and brought into the mitochondria, but when mitochondrial levels of GSH are deficient in comparison to mitochondrial ROS levels, cells undergo apoptosis, ferroptosis, necroptosis, and other cellular dysfunction [53].

**Figure 3:**
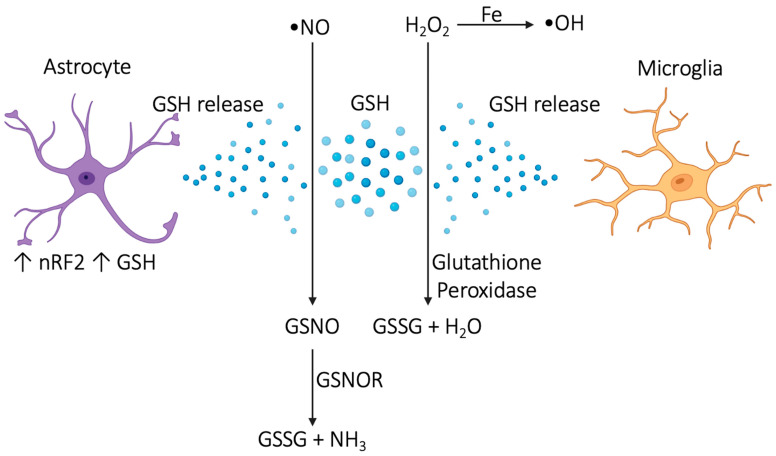
Astrocytes and microglial cells release GSH, which is involved in the reduction of ROS and RNS into safe metabolites.

In general, GSH protects against oxidative stress by turning a toxic ROS or RNS into something that can be safely dealt with. For example, GSH is used to convert H_2_O_2_ into H_2_O instead of allowing H_2_O_2_ to interact with iron via the Fenton reaction and become a harmful hydroxyl radical [54]. When nitric oxide (NO) reacts with any ROS, RNS are created. RNS are created in large part as a byproduct of mitochondrial respiration in the liver. When reactive nitric oxide interacts with GSH, S-nitrosoglutathione (GSNO) is formed. GSNO is further reduced into GSSG and NH3 by S-nitrosoglutathione reductase (GSNOR); see Figure 3.

The antioxidant nature of GSH is the primary reason it is used as a treatment. Conversely, deficient GSH levels allow for oxidative damage. In HIV, for example, the low levels of serum GSH measured by ELISA seen in patients with untreated HIV are associated with worse outcomes, while the initiation of ART results in increased levels of CD4+ T cells, increased levels of GSH, and dramatically improved outcomes [55].

GSH provides neuroprotection to the CNS through the neutralization of oxygen radicals as previously described, but also through the chelation of heavy metals and through the regeneration of other antioxidants in addition to other mechanisms. Some of the heavy metals chelated in the CNS by GSH are mercury and lead. Some of these regenerated antioxidants include vitamin C, vitamin E, alpha lipoic acid, and coenzyme Q10.

Another mechanism by which glutathione aids in protection against oxidative damage is through the stabilization of inflammatory cytokines. In a study conducted by our lab in 2017, 30 individuals given L-GSH with CD4+ counts below 350 for 3 months had increases in IL-2, IL-12, IFN-γ; decreases in IL-6, IL-10, and free radicals; and stabilization in levels of TGF-β, IL-1, and IL-17 compared to their placebo counterparts [56].

### 3.3. Routes of Delivery

When considering glutathione as a treatment option, it is important to consider the effects of different routes of delivery. Orally administered GSH is broken down by gastrointestinal peptidase rather quickly and is not a particularly useful route of administration. IV GSH is broken down by serum γ-GGT within 7 min and is less useful for this reason [57]. Intranasal GSH has been found to increase levels of GSH within the brain [58]. GSH crosses the blood–brain barrier so slowly that a rate has not been able to be determined at any given saturation and has generally been considered to be unable to directly cross it [59]. However, liposomal drugs, including liposomal GSH (L-GSH), can cross the blood–brain barrier via adsorptive-mediated transcytosis (AMT), receptor-mediated transcytosis (RMT), and carrier-mediated transports (CMT) [60]. One of the newer methods of increasing brain GSH levels is being researched and involves using ultrasound combined with microbubbles containing anti-miR-96-5p [61].

There are no studies measuring brain GSH levels after the administration of L-GSH. We will instead review a few studies that have measured the efficacy of L-GSH in other parts of the body, organized by route of the body. In a study of 12 subjects in which some were given oral glutathione tablets were found to have increased serum GSH compared to their placebo counterparts [62]. In another study, 11 subjects were given oral L-GSH solution while 7 controls were given placebos. After 3 months of treatment, blood was drawn and infected with *Mycobacterium* in vitro. An analysis of participants’ blood and granulomas formed showed the maintenance of serum GSH levels and reduction of *Mycobacterium* colonies within the granuloma as compared to those in the placebo group [63]. Although sublingual L-GSH has not been used in many research studies, sublingual GSH seems to be more efficacious than oral routes and was able to raise vitamin E levels when neither NAC nor oral GSH were able to within the same 12 individuals [64]. One study of 16 men found administration of sublingual L-GSH was associated with a reduction in LDL while the placebo group was not [65].

A separate mechanism for increasing brain GSH levels involves GSH prodrugs (e.g., NAC), which utilize high levels of peripherally administered cysteine to cross the blood–brain barrier via the L-amino acid uptake system [66]. Cysteine will then act as a substrate in glutathione synthesis within the brain, resulting in increased levels of GSH. The effectiveness of these prodrugs also depends on the route of administration. For example, oral NAC does not increase brain GSH, but IV NAC does (in one study, by 55% in patients with Parkinson’s disease) [67].

## 4. Glutathione in HIV-Associated Dementia (HIV Encephalitis)

HIV-associated dementia (HAD), the most advanced form of HAND, remains rare, with prevalence estimated to be between two and five percent of HIV patients [2,3,4]. It is a subcortical dementia affecting mood, memory, attention, and psychomotor function that differs from other dementias due to its mechanism of injury. HIV-infected monocytes release HIV proteins that can cross the disrupted blood–brain barrier in addition to the infected monocytes. Infected perivascular macrophages cause surrounding microglia to form nodules around the macrophages (giant cells), which results in the necrosis of the brain parenchyma (HIV encephalitis).

Treatment of HIV encephalitis involves initiating ART while avoiding efavirenz, which has the side effect of cognitive impairment. Other treatment options have been suggested in the past (2010), such as minocycline, memantine, and selegiline [68], but many have not been proven to be beneficial [68,69,70,71]. Patients respond well to ART therapy, although some individuals, especially young PLWH with otherwise good health, may experience minimal improvement of mild symptoms, such as mild cognitive impairment, while the same age category with severe symptoms, such as HIV encephalitis, tends to respond well to ART [72,73,74]. Unfortunately, microglial-based inflammation continues even after 4 years of ART [75]. Glutathione may be an option to help with mild cognitive deficits or as an option to improve outcomes in those on ART.

Since ART use has become more widespread, the frequency of HIV encephalitis caused by out-of-control HIV replication within the brain has drastically decreased. However, a new type of encephalitis has arisen in frequency: CD8+ encephalitis. This encephalitis presents with an acute onset of headache, encephalopathy, seizure, or coma and more commonly affects females. CD8+ encephalitis is a result of an autoimmune attack on the brain via CD8+ T cells, and it is more likely to result in death than HIV encephalitis [76,77,78]. In CD8+ encephalitis, perivascular white matter lesions and other diffuse white matter changes with possible cerebral edema are seen on most MRIs [78]. The 30% of patients that do not survive tend to die within 6 months, with mortality improved by corticosteroid treatments [76]. It is very hard to say what impact glutathione may have on CD8+ encephalitis, given that the mechanism revolves entirely around increased intracranial levels of CD8+ and that very little is known of glutathione’s effect on CD8+ levels in this situation.

There are no trials of glutathione supplementation for the treatment of HIV encephalopathy. In a study using melatonin as a treatment for encephalopathy, GSH levels were found to be elevated in hypoxic areas of the brain as a natural response, and melatonin did not further elevate those levels [79]. Melatonin typically increases GSH levels and has been proposed as one of the alternative treatments for neonatal encephalitis instead of or in addition to therapeutic cooling [80,81]. The increase in GSH seems to be a response to increased oxidative stress secondary to encephalopathy. We anticipate glutathione may be useful as an addition to ART because it has neuroprotective properties (as described in Section 3.2, although much of the mechanism is unknown). In mice with increased neuronal GSH (achieved by altering a glutamate/cysteine transporter), there was less oxidative damage to the hippocampus, according to fluorescent studies and immunohistochemical studies [82]. Although this study used gene targeting to increase levels of neuronal GSH, we know that we can use liposomal GSH (among other methods) to cross the blood–brain barrier and deliver glutathione to the brain instead [83]. Any future studies that trial glutathione as a treatment option would be wise to monitor the particular strains and clades with which HIV individuals are infected, as HIV-1 clade B leads to a greater reduction in GSH/GSSG than HIV-1 clade C [84].

## 5. Glutathione in HIV Peripheral Neuropathy

Peripheral neuropathy is the most common neurologic disease affecting patients with HIV, with as many as 35% of all people living with HIV being affected [85]. Patients most commonly have symmetric distal sensory neuropathies due to HIV and direct and indirect neurotoxicity from ART. Patients also have reactive inflammatory responses and vascular inflammation when starting ART therapy, which causes nerve damage in many patients [86]. Patients less commonly have acute inflammatory demyelinating polyradiculoneuropathy (AIDP), chronic inflammatory demyelinating polyradiculoneuropathy (CIDP), mononeuropathies, cranial neuropathies, autonomic neuropathies, radicular myelopathies, and amyotrophic-lateral-sclerosis-like motor neuropathy [87]. Since it is hard to distinguish whether HIV or ART is causing these neuropathies, different antiretrovirals are tried to alleviate symptoms.

Patients may also receive a treatment typically used for non-HIV neuropathies. Many of the typical peripheral sensory neuropathy treatments, such as tricyclic antidepressants [88,89] and pregabalin [90], have been found to be ineffective in HIV-related sensory neuropathies (HIV-SN). In a study of just 15 patients receiving gabapentin compared to 11 controls, gabapentin was found to be significantly more effective than a placebo for HIV-SN [91]. Some of the newer options being investigated are immunomodulation (e.g., IVIG, plasmapheresis) and anti-CMV therapies (e.g., ganciclovir) [92]. Neuropathies have different causes and respond differently to treatment options. The same fact remains for the subsets of HIV neuropathy. For example, IV ganciclovir tends to be effective for progressive polyradiculopathy (PP) in HIV patients because it tends to be caused by CMV. The most common neuropathy, distal sensory polyneuropathy (DSP), is usually a result of a medication side effect and almost never a result of CMV.

Although there are many options for treating HIV-associated peripheral neuropathies, none of them utilize the same mechanism as glutathione. Liposomal glutathione supplementation may provide further benefit to the inflammatory-mediated peripheral neuropathies seen in HIV patients, just as it benefits those with chemotherapeutic peripheral neuropathies, which often act through reactive oxygen species [93,94,95,96]. Although the mechanism was not tested or studied in these trials, L-GSH may be useful because it protects against ROS.

For many with HIV peripheral neuropathy, symptoms do not improve with typical treatments. Atypical treatments, such as cannabis, recombinant human nerve growth factor, and topical capsaicin, each have their own limitations (whether legal, financial, or efficacious) [97]. As a result, L-GSH may be a useful therapy to consider in these patients as well.

## 6. Glutathione in Lymphomas

Up to 40% of patients with HIV will get cancer [98]. Lymphomas are the most common cancers that cause death in patients with HIV. A total of 2% of all patients with HIV will face a lymphoma within their lifetime [99]. Additionally, patients with AIDS tend to face higher-grade lymphomas than those without AIDS [100]. Each of the lymphomas seen in patients with AIDS (Hodgkin lymphoma, Burkitt lymphoma, diffuse large B cell lymphoma (DLBCL), plasmablastic lymphoma (PBL), primary central nervous system lymphoma (PCNSL), and primary effusion lymphoma (PEL)) have different treatment regimens and overall survival rates. Apart from PEL and PCNSL, overall survival is greater than 70%. Overall survival for PEL and PCNSL is 40% and 60%, respectively [98]. Most treatments, however, involve different ART and chemotherapy combinations unless there is significant concern for drug–drug interactions.

Some lymphomas are so rare that they do not have a standard chemotherapeutic regimen. For example, plasmablastic lymphoma (PBL), one of the most deadly lymphomas, has a median overall survival of 58.6 months even while on multiagent chemotherapy [101]. Furthermore, 50% of patients with PBL are immunocompromised, but HIV status had no effect on survival rates. For patients with lymphoma facing poor outcomes, we feel it is worthwhile to investigate all potential options.

It is overall well understood that GSH plays a role in the initiation of cancer. Cells without GCLM (a modifier subunit of glutamate cysteine ligase) do not as readily undergo malignant transformation as their unaffected counterparts [102]. Later in cancer development, other antioxidant pathways, such as the thioredoxin antioxidant pathway (TXN), can take over the role of continuing to develop the cancer. If both the GSH antioxidant pathway and the thioredoxin antioxidant pathway are blocked, the transformation of the cancer ceases [102]. We can measure these effects by utilizing certain drugs. Buthionine sulfoximine can inhibit the GSH pathway. Sulfasalazine and auranofin can block the TXN pathway [102]. We could easily conclude that all cancers will have elevated intracellular levels of GSH; however, each cancer is different, and serum GSH levels are actually low in individuals with brain and liver cancers [103]. In addition, the biological composition of these lymphomas may differ depending on whether the patient is using ART or not [99,104].

The use of glutathione or glutathione prodrugs in chemotherapy is likely to be very nuanced. This is especially complicated in patients with HIV since the biological composition of these lymphomas may differ depending on whether the patient is using ART or not [99,104]. For example, ART can bring back immune functioning, allowing the host to kill some lymphoma cells more easily than other lymphoma cells, dependent on the host’s immunologic history. Part of a typical tumor’s protective defense against chemotherapeutic agents is increasing intracellular GSH levels so that more GSH can bind to the xenobiotic and utilize the mercapturic acid pathway as a means of eliminating the chemotherapeutic [105]. In most situations, inhibiting GSH activity increases the power of chemotherapeutic agents. This is usually beneficial, but in some drugs that cause significant toxicity beyond the tumor, GSH depletion is problematic. Doxorubicin, for example, is known to cause significant cardiotoxicity. Part of the cause seems to be due to “futile redox cycling”, for which glutathione plays an extremely important protective role [106]. It is for this reason that different modulations of doxorubicin have been made, including liposomal doxorubicin, pegylated doxorubicin, and glutathione-decorated pegylated doxorubicin [107].

## 7. Glutathione in Neurosyphilis

Neurosyphilis affects somewhere between 17.5 and 23.5% of patients with HIV [108,109,110]. The treatment is typically IV penicillin G for 10–14 days (or IM penicillin G and PO probenecid for 10–14 days). Symptoms depend on the degree of advancement of syphilis prior to treatment. Early neurosyphilis may be neurologically asymptomatic or may have meningeal symptoms (headache, nausea, vomiting, neck stiffness, cranial nerve deficit, seizure) or meningovascular symptoms (stroke-like symptoms secondary to cerebral thrombus or spastic weakness with muscle atrophy and sensory loss secondary to spinal cord vessel infarct). Late neurosyphilis is characterized by general paresis and tabes dorsalis.

Since treatment for this potentially debilitating condition is so effective and so widely available, there has been little reason to investigate the potential benefit of glutathione in the treatment of neurosyphilis. Even patients that go on to have a Jarisch–Herxheimer reaction are well managed with acetaminophen and have a resolution of symptoms for 24 h.

However, high doses of penicillin are known to inflame the liver, resulting in elevated liver enzymes. This mechanism is not fully elucidated but involves at least ROS and likely reactive metabolites somewhere along the cascade of effects. GSH has been used to protect the liver from oxidative stress in situations causing elevated liver enzymes. Its mechanism in the setting of penicillin-induced liver inflammation and injury has not been fully uncovered, although it seems to involve protection against ROS. A 2017 trial of 34 patients with NAFLD showed that glutathione reduced ALT by 13% compared to baseline [111]. We do not expect GSH to worsen the neurosyphilis condition since neurosyphilis infection is correlated with increased IFN-γ and IL-17 levels, which serves as evidence of CD8+ T-cell response to the spirochete. Our lab has found that glutathione helps stabilize the levels of these cytokines [56].

## 8. Glutathione in Secondary Viral, Fungal, and Parasitic Brain Infections

There are no completed trials of glutathione being used to treat any brain infections, whether viral, fungal, or parasitic. The viral infections are Progressive Multifocal Leukoencephalopathy, Herpes Simplex Virus Encephalitis, Cytomegalovirus encephalitis, Varicella Zoster Virus encephalitis, and HIV encephalitis. The fungal infections are cryptococcal meningitis, cerebral aspergillosis, histoplasmosis, coccidioidomycosis, and candidiasis. The parasitic infections are Toxoplasmosis, Cerebral Malaria, Trypanosomiasis, and Acanthamoeba encephalitis.

For each of the aforementioned infections, the mechanism of action that glutathione may utilize will be similar. In each infection, a pathogen initiates at minimum localized inflammation, noting that viral proteins can travel much further, thus spreading inflammation further. The presence of the pathogen induces immune responses that create ROS that will need to be managed by astrocytes and microglia, both of which require adequate GSH to handle the metabolites of the ROS. Each of the fungi or parasites will cause the dysregulation of important neurotransmitters. They may also affect the function of astrocytes and microglia directly. The result will be high or low levels of glutamate, which will require the astrocyte and astrocytic GSH to stabilize. L-GSH may be useful in supporting these cellular processes and offering neuroprotection against these pathogens. These are purely theoretical and simplistic evaluations of complex interactions. We aim only to communicate that there is some theoretical possibility that glutathione may be useful in parasitic diseases in HAND. Further studies are necessary to make any meaningful conclusions.

We will specifically discuss cryptococcal meningitis and tuberculosis (TB) in this section since they are the two most deadly diseases within this category. 

Patients with both HIV and TB are usually started on antiretrovirals and anti-tubercular drugs. Our lab has previously found that increasing intracellular glutathione stores with N-acetylcysteine resulted in decreased levels of IL-1, IL-6, and TNF-α, and increased levels of IFN-γ, as well as improved immune responses to *Mycobacterium tuberculosis* [112]. The study used human monocyte-derived macrophages from 20 subjects subsequently infected with *M. tuberculosis* in vivo. The amplified response of macrophages allowed the host to decrease the replication of *M. tuberculosis* [113]. Patients with concurrent antiretroviral and anti-tubercular regimens are most at risk for developing immune reconstitution inflammatory syndrome (IRIS). In patients with TB, ART increases CD4+ cell counts and immune response to the TB [114]. In IRIS, the immune response is overly robust and causes injury to the host. Patients with IRIS will have increased malondialdehyde levels, increased Th17 cell counts, decreased superoxide dismutase levels, and decreased Treg cell counts [115]. Our lab also found that glutathione seems to stabilize Th17 cell counts, evidenced by stabilized IL-17 levels after liposomal glutathione supplementation [56].

Cryptococcal meningitis is the second largest cause of death in patients with HIV after tuberculosis (TB), causing about 19% of AIDS-related deaths annually [116,117]. It tends to be more acute and more severe in patients with HIV. Treatment is amphotericin B with flucytosine followed by maintenance on fluconazole. Patients tend to present with severe headaches, nuchal rigidity, and papilledema. Unfortunately, patients still die despite the treatment regimen, so further research is always ongoing regarding this prevalent disease. As in many infectious diseases, ferroptosis is being proposed as one of the potentially effective mechanisms. Ferroptosis is dependent on low GSH levels [118], and GSH supplementation is likely not beneficial with any drug that relies on this mechanism.

## 9. Glutathione in Vacuolar Myelopathy

HIV-associated vacuolar myelopathy (HIVM) is the most common spinal cord disease in HIV and AIDS patients [119]. In an autopsy-based case–control study, 46.5% of AIDS patients were diagnosed with vacuolar myelopathy, but symptoms only manifested in those with advanced disease [120]. Many individuals with vacuolar myelopathy may not show signs or symptoms until their death. In this study, 56 of the individuals had prior neurologic evaluations that were utilized to make determinations regarding symptom presentation and the stage of the disease discovered during autopsy.

HIVM presents as a slowly progressive spastic paraparesis with sensory abnormalities and gait dysfunction. Patients will also have concomitant HIV-associated dementia since it most commonly shows up in the late stages of HIV. HIVM is due to vacuolization of the lateral and posterior columns of the spinal cord, typically at the thoracic levels, resulting in sensory issues below the level of the lesions (i.e., affecting lower extremities). As the condition is rare and hard to diagnose at an appropriate time due to its similar presentation to the subacute combined degeneration seen in B12 deficiency, case reports provide valuable information. In one case report of a 34-year-old man who experienced cognitive changes, spasticity, gait dysfunction, and urinary incontinence, the initiation of ART resulted in improved cognitive changes but no improvements to spasticity or urinary incontinence [121]. A case report in India in 2020 described the treatment of vacuolar myelopathy with IVIG, resulting in the resolution of spastic paresis and sensory processing disorders with a retained Babinski’s sign [122]. Since the suspected success of IVIG for HIVM is thought to be attributed at least partly to addressing proinflammatory cascades, glutathione can be considered as a cheaper or more available option in trials as providers continue to look for any effective treatment for HIVM.

## 10. Glutathione in Psychological Conditions Related to HIV

A range of neuropsychiatric diseases are prevalent among people living with HIV, including but not limited to depression (22–50%), anxiety disorders (2–40%), PTSD (30%), substance use disorder (40–74%), sleep disturbance (10–50%), and severe mental illness such as psychosis (0.2–15%) [123,124]. The prevalence of severe and chronic mental illness, such as schizophrenia and bipolar disorder, is estimated to be from 4 to 19% [125]. Mania may occur in HIV patients as a component of a pre-existing bipolar disorder diagnosis and independently as a standalone condition known as AIDS mania. AIDS mania causes a form of manic episodes, which is unique to late-stage HIV infection [126,127].

Chronic stress and high HPA axis activity, as seen in individuals with depression and anxiety, are correlated with reduced GSH levels on the cortical surfaces of the brain as well as in the prefrontal cortex on magnetic resonance spectroscopy (MRS) [128,129,130,131]. Most anti-depression medications do not directly affect the oxidative stress that the brain encounters during psychogenic stressors but instead do so by increasing levels of GSH [132]. The mechanisms and effects of anxiolytics are even less well understood, and it is unclear whether L-GSH may help or harm the condition. In an 8-week trial involving just 20 patients, treatment with S-adenosylmethionine, a glutathione prodrug, resulted in an acute reduction in depressive symptoms as measured by the Hamilton rating scale for depression and the Beck Depression Inventory.

The mental state may play a role in pain perception in patients with HIV as well, with 80% of a 30-person cohort reporting improved pain after 3 months of mindfulness [133].

### 10.1. Post-Traumatic Stress Disorder (PTSD)

Pro-inflammatory states have been thought to be part of the process involved in patients with PTSD. In a meta-analysis of 44 studies, no consistent pattern involving anti- or pro-inflammatory cytokines was found [134].

### 10.2. Substance Use Disorder

It is well known that substance use results in oxidative damage to the heart, kidneys, and liver (among other organs). Patients with chronic Alcohol Use Disorder with HIV experience a synergistically increased oxidative stress compared to patients with either one of the conditions alone [135]. Glutathione plays a protective antioxidant role, and in situations of substance use, where the antioxidant role may be overpowered, L-GSH will likely improve outcomes.

Methamphetamines impair astrocytes by internalizing connexin 43 and reducing the expression of connexin 36 in astrocytes [136]. HIV also impairs astrocytes, and although methamphetamines cause astrocytes to release more glutathione reductase, which should have an antioxidant effect, oxidative stress is still seen [136].

Unfortunately, there were no other relevant studies reviewed pertaining to substances abused and glutathione or other very similar treatments.

### 10.3. Sleep Disturbance

There is not necessarily a clear link between the disease process of HIV and sleep disturbances. Still, 70% of PLWH experience sleep disturbances [137]. For the sake of addressing this symptom that may or may not be related, it has been included in this text.

Glutathione supplementation has recently been proposed in the media as a melatonin-like sleep aid (usually mentioning its antioxidant properties that repair your body while asleep). The literature does show that sleep deprivation results in oxidative stress [138,139], but none of the literature is concerned with the effect of glutathione on subjective sleep quality or objective sleep architecture in humans. One study involving 12 mice found N-acetylcysteine decreased sleep-onset latency and increased time spent in NREM per intracranial EEG compared to mice receiving saline [140]. Human trials with liposomal glutathione would shed more light on its potential efficacy for patients with sleep disturbances, including those with HIV who are likely to have sleep disturbance secondary to a process involving oxidative stress.

### 10.4. Psychosis

Few mechanisms leading to psychosis have been alluded to, and, for the most part, psychosis remains mysterious. Some of the leading theories involve a lack of communication or connectivity between eloquent brain structures. Nine patients with early psychosis were treated with NAC for 6 months and found to have increased connectivity per EEG compared to those receiving a placebo [141]. A previous study by the same group found that administering NAC for 6 months led to statistically significant increases in GSH levels, averaging around 23% in the medial prefrontal cortex [141]. It is likely that liposomal glutathione supplementation will show similar benefits.

### 10.5. AIDS Mania

AIDS mania is a mania that can present at any stage of HIV. It is currently considered one of the potential ways that AIDS may present. However, it is treated exactly like all other manias and responds extremely similarly. There is likely no benefit to using glutathione since it would only provide an antioxidant effect, and lithium does this as well.

## 11. Conclusions

There is very little research performed on the direct effects of glutathione supplementation in HAND. We feel there is great potential for research in this area. It is evident, based on our findings, that the diseases will need to be investigated individually, very carefully, due to the great variability seen in mechanisms within each of the HIV-associated neurocognitive diseases.

## Data Availability

Not applicable.
